# Physiological and behavioral response of the Asian shore crab, *Hemigrapsus sanguineus*, to salinity: implications for estuarine distribution and invasion

**DOI:** 10.7717/peerj.5446

**Published:** 2018-08-14

**Authors:** David M. Hudson, D. Joseph Sexton, Dinsdale Wint, Connor Capizzano, Joseph F. Crivello

**Affiliations:** 1 Department of Research and Conservation, The Maritime Aquarium at Norwalk, Norwalk, CT, United States of America; 2 Department of Physiology and Neurobiology, University of Connecticut, Storrs, CT, United States of America; 3 Department of Biology, Georgia State University, Atlanta, GA, United States of America; 4 Momenta Pharmaceuticals, Cambridge, MA, United States of America; 5 School for the Environment, University of Massachusetts at Boston, Boston, MA, United States of America

**Keywords:** Salinity tolerance, Invasiveness, Crustacean, *Hemigrapsus*, Survival curve, Invasive species

## Abstract

The invasive Asian shore crab, *Hemigrapsus sanguineus*, is ubiquitous in the rocky intertidal zone of the western North Atlantic. A likely contributor to this colonization is that *H. sanguineus* is able to handle a wide range of salinities, and is thus more likely to spread through a greater geographic area of estuaries. This study investigated the salinity effects on this animal by observing survival across a range of salinities, the maintenance of hemolymph osmolality under different salinities, and behavioral preference for and avoidance of salinities. *H. sanguineus* showed high survival across a broad range of salinities, had little change in hemolymph osmolality over a short-term salinity shock, and behaviorally distinguished between salinities when presented with a choice, under both acclimation salinities of 5 PSU or 35 PSU. Such results suggest *H. sanguineus* has a hardiness for the rapid changes in salinity that happen in the intertidal zone, yet is capable of physically moving to a more optimal salinity. This enhances their competitiveness as an invader, particularly surviving lower salinities that present challenges during high-precipitation events in rocky intertidal areas, and partially explains this species’ dominance in this habitat type.

## Introduction

The invasive Asian shore crab, *Hemigrapsus sanguineus*, is a particularly successful invasive decapod crustacean species which is now found in estuaries and open coasts in areas along the western North Atlantic and western Europe, displacing resident species (Lohrer et al., 2000; Brousseau et al., 2002; Van den Brink, Wijnhoven & McLay, 2012; Landschoff et al., 2013; Gothland et al., 2013; Gothland et al., 2014). The species has become the most abundant crab in the rocky intertidal in New England since it was first found in New Jersey in 1988 (McDermott, 1998; Williams & McDermott, 1990; Lohrer & Whitlatch, 2002; Kraemer et al., 2007; O’Connor, 2014). Previous work in this lab and by others investigated the behavioral response of the intertidal and subtidal community to this species’ presence (Epifanio, 2013; Hudson, Reagan & Crivello, 2016). Conspecific tolerance also enhances its success in overcoming resistance to invasion (Hobbs, Cobb & Thornber, 2017). However, beyond community interactions, this species’ broad salinity tolerance could be contributing to its success as an invader and for the invasiveness of the genus more broadly (Tsai & Lin, 2007; Urzúa & Urbina, 2017), so this work aimed to evaluate its survival and behavior with respect to salinity.

Salinity is of particular importance in the marine environment to delineate biotic zones in estuaries. Organisms differ along a broad spectrum in their abilities to handle salinity changes, with fishes and macroinvertebrates (including crabs) proposed to inhabit five or six biotic salinity zones (Bulger et al., 1993; Wolf et al., 2009). Salinity tolerance could therefore be used in the management of resources in the context of locational risk for invasion by a particular species.

Invasive crab species from estuarine systems often have broader salinity tolerances to withstand rapid changes in salinity common in their native locales that are predictive for their success in new systems (McGaw & Naylor, 1992b; Colnar & Landis, 2007; Fowler, Gerner & Sewell, 2011). Salinity tolerance and preference is clearly important in determining invasiveness to intertidal zones in estuaries, particularly in decapods, and sheds light on potential areas they can invade successfully. Other notable worldwide invasive decapod crustacean species that draw attention to this particular salinity tolerance character include the Chinese mitten crab, *Eriocheir sinensis*, which spends much of its adult life in freshwater, but its larvae require full-strength seawater to survive (Rudnick et al., 2005). In addition, the Harris mud crab, *Rhithropanopeus harrisii*, native to eastern North America, is found in salinities down to 0.1 PSU and is establishing itself in new areas (Reisser & Forward, 1991; Roche et al., 2009; Kotta & Ojaveer, 2012; Fowler et al., 2013). Much of the work to determine osmoregulation in crabs was initially performed on a potent worldwide intertidal/estuarine invader, the European green crab, *Carcinus maenas* (Towle & Kays, 1986; Cieluch, 2004). Crabs osmoregulate utilizing the posterior gill filaments (Koch, 1954; Burnett & Towle, 1990; Lucu & Towle, 2003), with far greater Na^+^/K^+^ ATPase transport proteins expressed in the posterior gill than in the anterior gill (Burnett & Towle, 1990; Koch, 1954; Neufeld, Holliday & Pritchard, 1980). This transporter’s role in osmoregulation in crabs and other crustacean species is well-established (reviewed in Lucu & Towle, 2003; Tsai & Lin, 2007). Utilizing the changes in hemolymph osmolality as a result of this transporter’s activity over time of exposure, along with behavior, can therefore be a useful determinant of the implications of salinity change in the whole animal.

*H. sanguineus* experiences salinity stress below 15 PSU regardless of acclimation, indicated by increased heart rate and activity level (Depledge, 1984). The congener *Hemigrapsus crenulatus* shows increased oxygen consumption as salinity stress increases (as salinity decreases), strong hyper regulation at low salinities, with increases in regulatory capacity as crab size increases (Urzúa & Urbina, 2017). However, this species is easily exposed to salinities below 15 PSU during a freshwater event (i.e., rain, snow) in the intertidal zone. Tsai & Lin (2007) noticed little decrease in Na^+^/K^+^ ATPase activity in *H. sanguineus* between 5 PSU and 35 PSU treatments, while optima studies of congeners *H. crenulatus* (Urbina et al., 2010) and *Hemigrapsus takanoi* (Shinji et al., 2009) determined a 21 PSU optimum and 24.4 PSU optimum, respectively. Similarly, our previous initial gill work found no significant change in Na^+^/K^+^ ATPase activity in posterior gill of *H. sanguineus* when exposed to 35 PSU, 15 PSU, or 5 PSU seawater for 7 days, but did observe a short-term increase in activity at 2 and 4 h post-treatment for 15 PSU treatments (Hudson, 2011). Therefore, *H. sanguineus* has similar osmoregulatory ability with this transporter regardless of treatment, but may be able to increase its activity in the short term.

Species’ responses to gradients are particularly important in determining where they will fall within a physical range (Case & Taper, 2000), so a wider tolerance will mean a wider geographic footprint is possible. Specifically, salinity tolerance levels can greatly alter distribution of species along a coast (Teal, 1958; Barnes, 1967; Engel, 1977; Felder, 1978; Young, 1978; Young, 1979; Rabalais & Cameron, 1985; Hulathduwa, Stickle & Brown, 2007; Fowler, Gerner & Sewell, 2011; D Hudson, pers. obs., 2007). Freshwater events are common in estuarine areas, and salinity can also change on an hourly timescale with the tides, meaning that organisms living there must rapidly manage these challenges behaviorally and/or physiologically. Change in salinity is one of the most common forms of stress in the intertidal zone in estuaries, and several invasive crab species are known to be euryhaline in response (Reisser & Forward, 1991; Henry, Thomason & Towle, 2006; Roche et al., 2009; Fowler, Gerner & Sewell, 2011). This underscores the value of understanding how physiological capacity is related to behavioral choice or avoidance, since both contribute to the invasiveness of a species, i.e., how well a species reproduces and extends its range from its introduction point and starts populations in new places (Rejmánek, 2011).

To investigate this interaction between physiology and behavior, the work reported here includes physiological tolerance (i.e., maintenance of hemolymph ion concentration) and survival, but also incorporates the behavioral preference of *H. sanguineus* as an indicator of how well they can avoid risk. Since little change was detectable in gill physiology in previous work, a behavioral approach for this work investigated sublethal effects by quantifying behavioral avoidance and hemolymph osmolality change, along with investigating differences in overall survivorship over time. This tests the idea that *H. sanguineus* has an ability to tolerate wide salinity changes for a significant amount of time, and can also behaviorally avoid stressful salinities at small spatial scales, as have other species (Teal, 1958; Lagerspetz & Mattila, 1961; Jansson, 1962; Thomas, Lasiak & Naylor, 1981; Ameyaw-Akumfi & Naylor, 1987; McGaw & Naylor, 1992a; McGaw & Naylor, 1992b).

## Methods

Adult crabs with carapace widths between 15 mm and 34 mm were collected by hand off Avery Point in Groton, Connecticut, USA under Connecticut Department of Environmental Protection Scientific Collector’s Permits # SC-06040 and # SC-09015. Crabs were acclimated for at least 14 days in holding tanks at 35 PSU before use.

### Survival

A lab-based holding study was performed to evaluate the survival of *H. sanguineus* immersed in a broad range of salinity treatments typical for euryhaline species. Crabs were exposed to salinity treatments of 1 PSU, 5 PSU, 10 PSU, 15 PSU, or 35 PSU for 14 days, given the observed ability of the genus *Hemigrapsus* to tolerate low salinities for extended periods of time (McGaw, 2001; Tsai & Lin, 2007). The 1 PSU treatment, in particular, was included to simulate the nearly freshwater surface conditions during precipitation events in estuaries and tide pools. Specimens were kept in tanks at 20 °C that corresponded with spring and fall environmental conditions from the original capture location, Long Island Sound, including a 12-hour light/dark cycle. Crabs were held in groups, and cannibalism was accounted for as a cause of mortality if it occurred upon observation of mortality events, as were molt failures. Each salinity treatment consisted of 20 males and 20 females, which were fed with shrimp pellets every day to satiety. Crab survival was monitored daily over the course of the 14-day experimental trial where dead specimens were removed.

*H. sanguineus* survivorship over time (i.e., the survival function) was evaluated using methods traditionally used in the context of longitudinal survival analyses. Longitudinal data provide information on the time animals either died or were last observed alive due to ongoing monitoring of survival (Cox & Oakes, 1984; Benoît et al., 2015). Such longitudinal data for *H. sanguineus* consist of records for each crab specimen, which include information about the occurrence and timing of an event as well as salinity treatment values and sex that might affect survival (i.e., covariates). Crabs that were still alive when last observed or at the end of the experiment were treated as “right-censored” observations, for which their time of death was unknown either because mortality did not occur or was not observed during the holding period (Singer & Willett, 2003).

A set of non- and semi-parametric longitudinal analyses were first employed to evaluate the effect of salinity treatment and sex on the *H. sanguineus* survival function. The semi-parametric Cox proportional-hazards regression model was initially used given its ability to simultaneously evaluate the additive effect of multiple covariates (Cox, 1972). Preliminary regression model results suggested that the survival function was only dependent upon salinity (Table S1). Consequently, the non-parametric Kaplan–Meier estimator of survival was used to preliminarily identify if each salinity treatment produced distinct survival functions (Kaplan & Meier, 1958; Cox & Oakes, 1984; Fig. S1). The Kaplan–Meier estimator follows the proportion of individuals alive as a function of time in the absence of censored observations and is well-suited for univariate analyses with multiple factor levels.

The Peto & Peto modification of the Gehan-Wilcoxon was then used to accept or reject the null hypothesis that there was no statistical difference between survival functions (Harrington & Fleming, 1982). Multiple pairwise comparisons using the Peto & Peto test with Benjamini–Hochberg corrections to adjust for significance value inflation were subsequently applied to determine if and which salinity-dependent survival functions were statistically distinct from one another. Salinity-dependent survival functions that failed to reject the null hypothesis were subsequently combined. Preliminary results indicated survival was only significantly different between the 1 PSU and the 10 PSU, 15 PSU, and 35 PSU treatments (*p* < 0.01), and also between 5 PSU and 35 PSU (*p* < 0.05) (Table S2). However, no objective procedure could be performed to combine the survival functions with confidence given inconsistencies between pairwise comparison significance values (Table S2). For instance, while survival was not statistically different between the 1 PSU and 5 PSU as well as the 5 PSU and 10 PSU groups (*p* > 0.05), they could not be combined since survival between the 1 PSU and 10 PSU groups was statistically significant (*p* < 0.01). Coarser salinity categories were therefore examined and presented for easier interpretation of results, specifically fresh (1 PSU), estuarine (5–15 PSU), and seawater (35 PSU) salinity groups.

All survival-related analyses were performed using the statistical computing software R (version 3.4.2; R Core Team, 2017) with added functionality from the associated package “survival” (version 2.38; Therneau, 2015) and “survminer” (version 0.4.0; Kassambara & Kosinski, 2017). Statistical significance was accepted at a level of *p* < 0.05.

### Salinity preference

The behavioral preference of *H. sanguineus* for specific salinities was evaluated through a separate lab-based experimental trial with new specimens. This study utilized an arena that contained two 10 cm × 10 cm chambers, each with a different salinity and bubbled with an airstone, connected by an above-water bridge to offer a binary choice, consistent with past studies (Teal, 1958; Lagerspetz & Mattila, 1961; Jansson, 1962; Thomas, Lasiak & Naylor, 1981; Ameyaw-Akumfi & Naylor, 1987; McGaw & Naylor, 1992a; McGaw & Naylor, 1992b). *H. sanguineus* is a highly mobile crab that in initial trials actively ran back and forth between the chambers over the bridge, meaning that it was able to effectively sample the conditions of both chambers. Therefore, individual crabs could chose to either (1) stay in the initial chamber, (2) relocate to the second chamber by using the connecting bridge, or (3) remain on the bridge since these are intertidal crabs. Because this species exists in estuaries in the field and therefore along a broad salinity gradient, individual *H. sanguineus* were acclimated to either 5 PSU or 35 PSU for a period of 14 days prior to the experiment to test the effects of acclimation. Since these are poikilothermic animals, activity increases with temperature. As such, temperature effects on preference were quantified by acclimating specimens at either 10 °C or 20 °C at those same salinities to simulate seasonal water temperature differences and gauge the general capacity of the animals to behaviorally regulate during different seasons. An extended acclimation time of 14 days was used to account for longer exposure to lower salinities further up an estuary and during freshwater influx events, unlike the rapid changes (i.e., ∼6 h) that occur in the littoral zone. Given acclimation conditions have been shown to modify preference behavior in other crustacean species (Gross, 1957; Hernández et al., 2006), we investigated whether acclimation conditions (temperature and salinity) affected salinity preference. We determined whether specimens had any preference for a lower or higher salinity based upon the salinity and temperature during their acclimation period. Individual crabs were presented with pairwise choices between 5, 15, or 35 PSU for a period of 12 h, with final location at 12 h recorded, for 25 replicates for each sex and acclimation at two acclimation temperatures (total of ∼100 per salinity comparison). Due to the initial high activity of this crab species, the final location at 12 h was considered the “chosen” condition.

Behavioral choice of salinity data were analyzed for binary choice by chi-square test, and then the probability of leaving starting salinity was analyzed by one-way ANOVA for each of starting salinity, sex, acclimation salinity, and temperature. In order to test interactive effects between those four factors, multiple two-way ANOVAs were completed in the statistical computing software R (version 3.4.2; R Core Team, 2017).

### Hemolymph response to salinity change

To quantify hemolymph osmolality response to salinity shock, crabs were acclimated to full-strength seawater salinity (32 PSU) for 14 days to normalize gene expression (Tsai & Lin, 2007), then 40 specimens were exposed for seven days (168 h) to each of the following salinity treatments: 32 PSU (control), 17.5 PSU, 10 PSU, and 5 PSU. Five animals were taken out of the 32 PSU seawater after the 14-day acclimation period and used as the initial time point for all treatments. Due to high mortality in the survival study for some of the lowest salinities (Fig. S1), the experiment was only run for 7 days. Crab hemolymph was sampled from five new animals at each post-exposure time point of 1, 2, 4, 8, 24, 48, 72, and 168 h (7 days) and frozen at −80 °C. The early time points were chosen to compare with results for other crabs, as work in another euryhaline crab, *Callinectes sapidus*, supports little change in observed hemolymph osmolality values within 12 h (Sommer & Mantel, 1988; Towle, 1997; Henry et al., 2002) of salinity shock. Hemolymph samples were taken with the use of a 21 gauge syringe inserted into the crab’s branchial cavity, and stored at −80 °C in 1.5 mL centrifuge tubes. Samples’ hemolymph osmolality was measured after removal from thaw, centrifuged for 1 min at 10,000 rpm, and run in duplicate on a Wescor 5100C vapor pressure osmometer. Samples were run in duplicate, with the average of the two taken as the value for that sample. These results were then analyzed by two-way ANOVA for effects of exposure time and treatment, along with interactive effects between the two. A repeated measures ANOVA would be inappropriate to analyze hemolymph data, as the individuals were sacrificed at each time point for a separate study of the upregulation of proteins in posterior gill tissue. Each time point was analyzed for differences between the four salinity treatments by a one-way ANOVA with Tukey post-hoc analysis. Time zero was left out of analysis since it was the same for all four treatments. All statistics were completed in R statistical computing software (version 3.4.2; R Core Team, 2017).

## Results

### Survival

**Figure 1:**
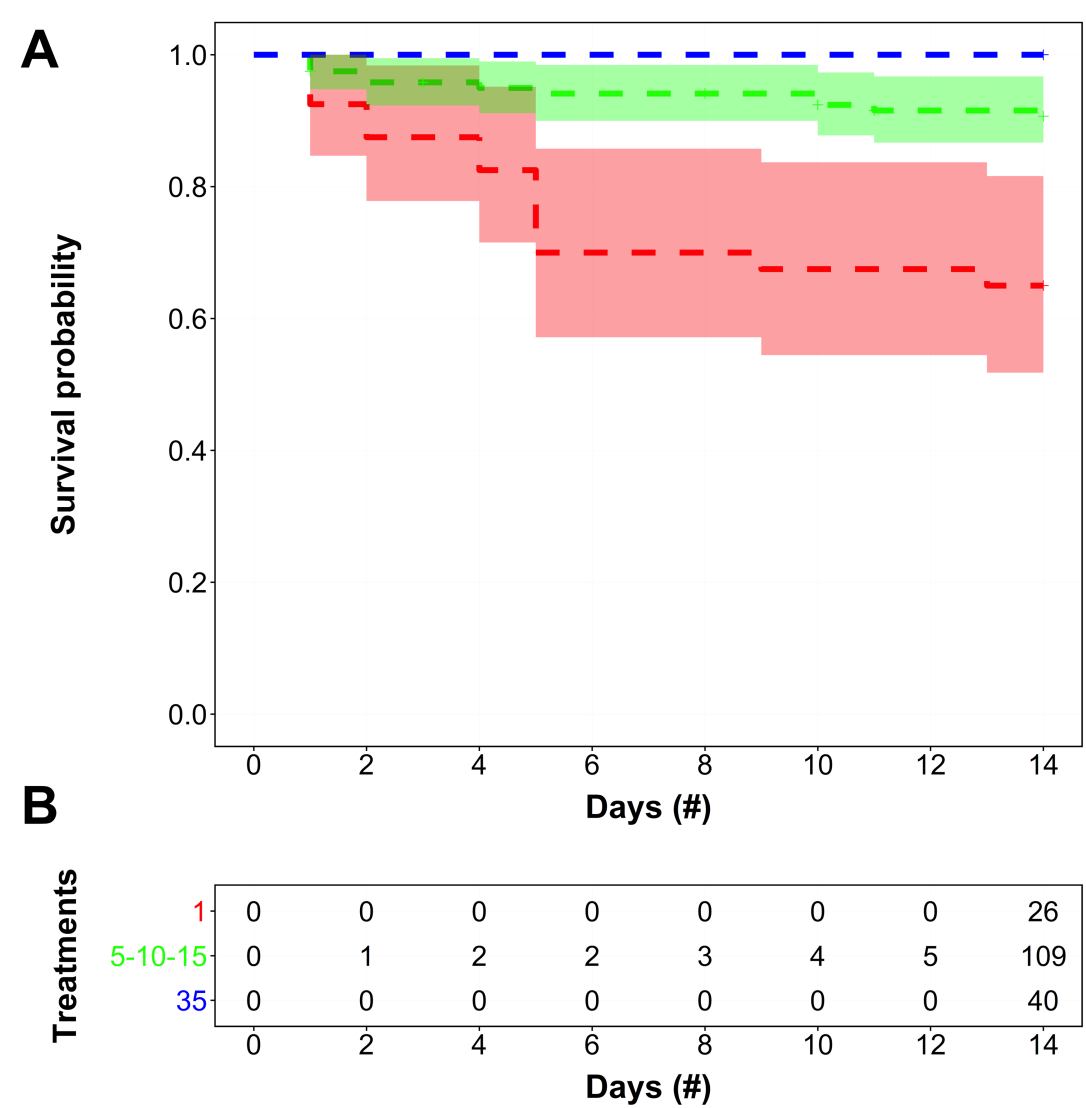
Survivorship over time of *H. sanguineus* in different salinities. (A) The survivorship over time is depicted as the survival function (dotted line), of *H. sanguineus* by broad salinity categories (1 PSU = red; 5–15 [5, 10, 15] PSU = green; 35 PSU = blue). Shaded areas represent the 95% confidence bands estimated for the salinity-specific non-parametric Kaplan–Meier survival functions. Plus symbols (+) on each survival function indicate when an individual was last observed alive (i.e., right-censored). (B) The frequency table tracks the cumulative number of right-censored individuals for each survival function over time.

The semi-parametric Cox proportional hazards regression model indicated that the *H. sanguineus* survival function was only dependent on salinity treatment with no effect from the sex covariate (Table S1). When survival data were grouped into broader salinity designations for ease of interpretation and applicability to representative scenarios, the non-parametric Kaplan–Meier estimator indicated that *H. sanguineus* survival functions for the fresh, estuarine, and seawater salinity groups were distinct (Fig. 1), which was reaffirmed by the Peto & Peto test against all three survival functions (*χ*^2^ = 26.8, *d.f.* = 2, *p* ≪ 0.001). Moreover, multiple pairwise comparisons between all broader salinity survival functions were statistically different, thereby confirming survival was distinct between groupings (Table S3). For instance, the difference in survival between the 35 PSU (highest survival) and pooled 5 PSU/10 PSU/15 PSU treatments (high survival but some mortality) was significant (*p* < 0.05). There were also significant survival differences between the 35 PSU treatment and the 1 PSU treatment (lowest survival rate) (*p* < 0.001), and between 1 PSU (lowest survival) and the pooled 5 PSU/10 PSU/ 15 PSU treatments (high survival but some mortality) (*p* < 0.001) (Table S3). Therefore, it seems that while there is clearly a difference between the highest and lowest salinity treatments, the middle three salinity treatments have a moderate survival rate that is significantly different from both the upper and lower salinity treatments. Interestingly, survival did not differ significantly among the three salinity groups over the first 7 days of observation (*p* = 0.421).

### Salinity preference

**Figure 2:**
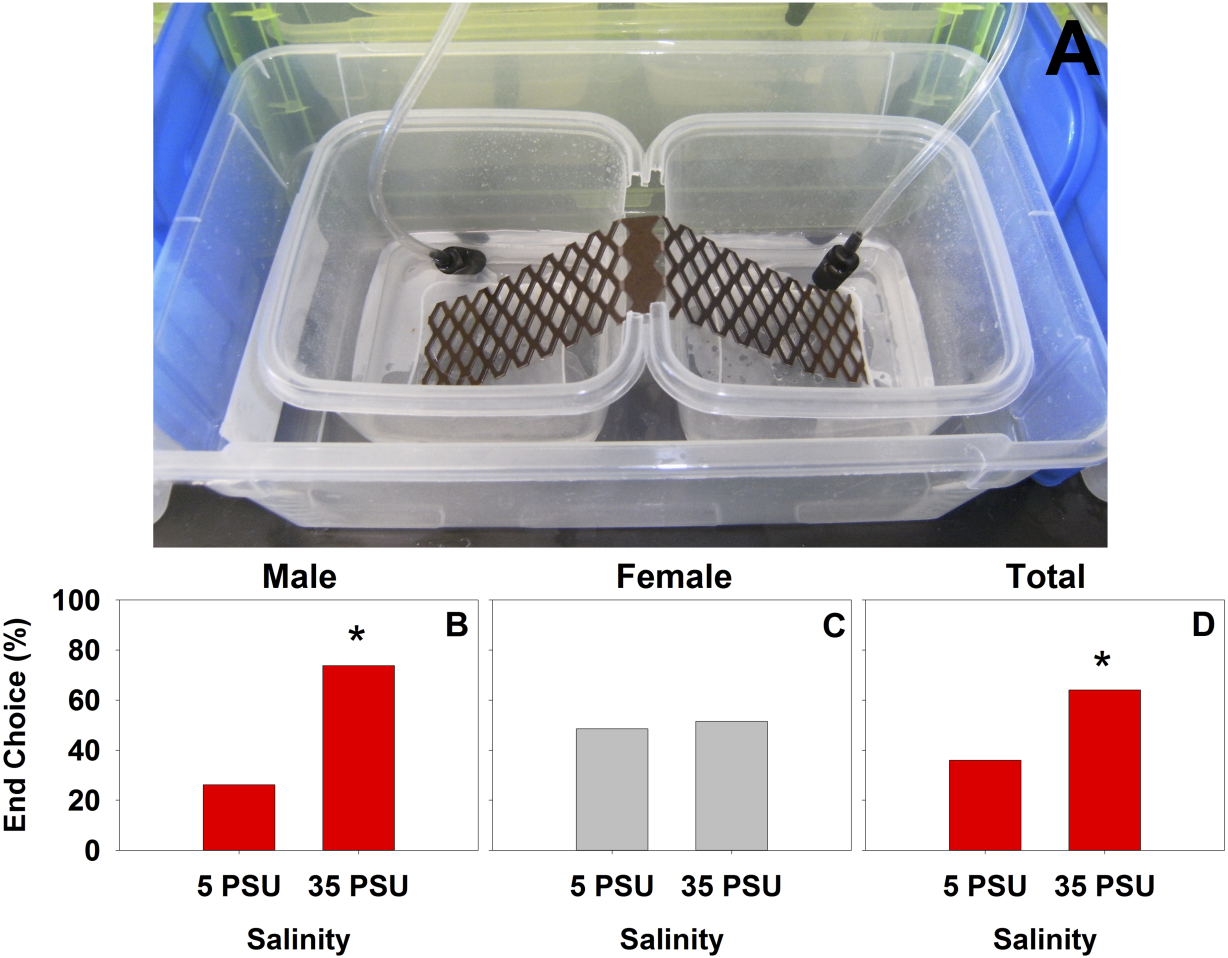
Salinity choice at the end of 12-hour experiments. The laboratory setup for choice is seen in (A) (photo credit David M. Hudson). The end choice after 12 h was only significantly different for the comparison of the most extreme salinities at 20 °C, which maintained significance for males (B), was not significant for females (C), and was significant overall (D).

Behavioral preference experiments indicated a significant preference (*χ*^2^ = 5.88, *d.f.* = 1, *p* < 0.05, *n* = 75) of *H. sanguineus* for 35 PSU over 5 PSU seawater at 20 °C regardless of acclimation (Fig. 2D), but no significant preference was exhibited when individuals were given a choice between 35 PSU and 15 PSU (*χ*^2^ = 0.653, *d.f.* = 1, *p* > 0.05, *n* = 75), nor for 5 PSU and 15 PSU PSU (*χ*^2^ = 1.174, *d.f.* = 1, *p* > 0.05, *n* = 69). This significance appears to come from two sources. Males at 20 °C (Fig. 2B) showed a significant preference for 35 PSU over 5 PSU (*χ*^2^ = 9.52, *d.f.* = 1, *p* < 0.01, *n* = 42), and also had a significant difference in preference towards 35 PSU when first acclimated to 35 PSU (*χ*^2^ = 7.2, *d.f.* = 1, *p* < 0.01, *n* = 20). Crabs that were acclimated to 5 PSU prior to the experiment chose 35 PSU over 5 PSU (*χ*^2^ = 4.8, *d.f.* = 1, *p* < 0.05, *n* = 30). Aside from these, there are no other significant effects of acclimation on final salinity choice.

**Figure 3:**
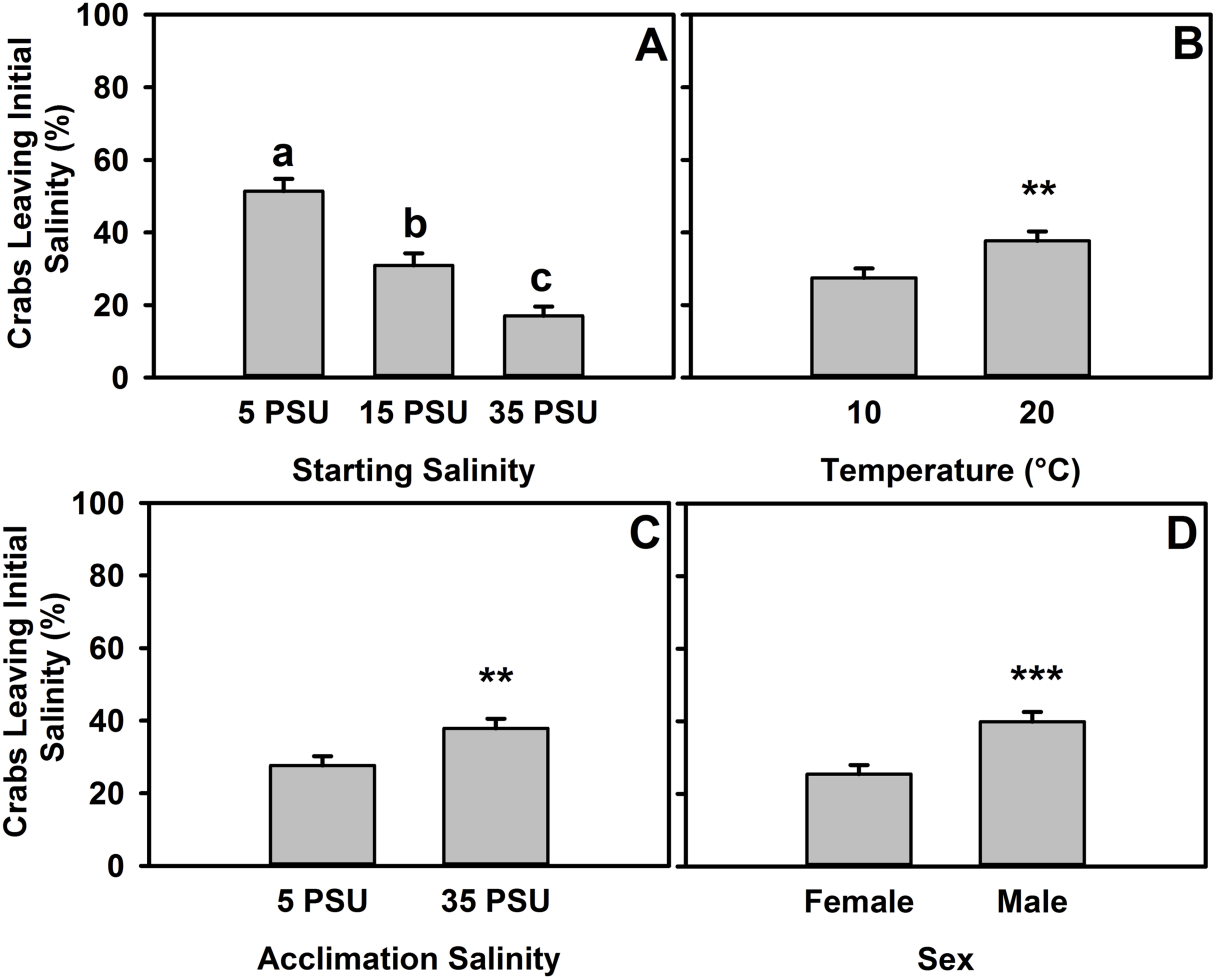
Percent of *H. sanguineus* leaving starting salinity after 12 hours. (A) Percent of crabs leaving the starting location as a function of starting salinity (5 PSU, 15 PSU, or 35 PSU). (B) Animals’ likelihood of leaving based on temperature of experiment, as pooled data across starting salinities. (C) Animals’ likelihood of leaving any starting salinity based on original acclimation salinity of the animal, as pooled data across all starting salinities. (D) Animals’ likelihood of leaving starting salinity analyzed by sex, as pooled data across starting salinities. All error bars are standard error.

These data were also analyzed by whether the crab left the starting salinity in any experiments (5 PSU, 15 PSU, or 35 PSU). There was a significant effect of starting salinity on whether crabs were more or less likely to leave (one-way ANOVA, *F* = 32.55, *d.f.* = 2, *p* ≪ 0.001, *n* = 635). Interactions between factors were not significant in the two-way ANOVAs used to determine interactive effects between acclimation salinity and starting salinity nor between acclimation salinity and sex (Tables S4 and S5, respectively), but the interaction between starting salinity and temperature was significant (Tables S6), and a trend exists for an interaction between sex and temperature (Table S7). Crabs that started in 5 PSU (at both temperatures), whether for 5 PSU × 35 PSU or 5 PSU × 15 PSU experiments, were more likely to leave that salinity (move to the other salinity, escape, or move onto the ramp) (Tukey’s post-hoc test, *α* = 0.05, *p* < 0.001) than those which started in 15 PSU or 35 PSU (Fig. 3A). Additionally, crabs that started in 15 PSU were more likely to leave than those in 35 PSU (Tukey’s post-hoc test, *α* = 0.05, *p* < 0.01). As the experiment was completed in both 10 °C and 20 °C (Fig. 3B), animals were 37.7% likely to leave a salinity at 20 °C, whereas at 10 °C it was 27.5% (one-way ANOVA, *F* = 7.475, *d.f.* = 1, *p* < 0.01, *n* = 635). Acclimation had a significant effect (Fig. 3C), with animals more likely to leave the starting salinity if they were acclimated to 35 PSU (one-way ANOVA, *F* = 7.585, *d.f.* = 1, *p* < 0.01, *n* = 635). There was an effect of sex on the likelihood that an animal would leave the starting salinity (Fig. 3D), with males more likely to leave at 39.9% and females leaving 25.4% of the time (one-way ANOVA, *F* = 15.26, *d.f.* = 1, *p* < 0.001, *n* = 635).

### Hemolymph response to salinity change

**Figure 4:**
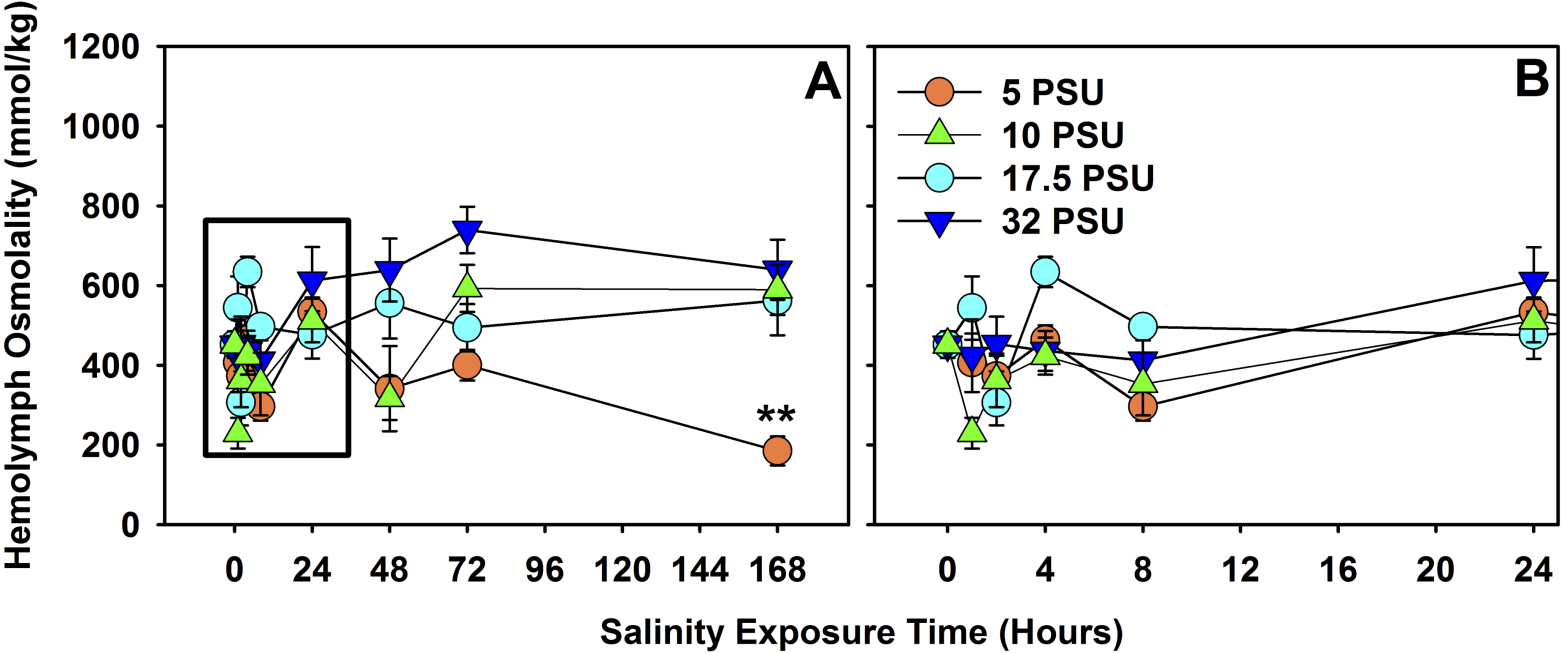
Hemolymph osmolality of *H. sanguineus* after 7 days of exposure to different salinities. (A) Total 7-day exposure results of hemolymph osmolality response to the four treatment salinities, 32 PSU, 17.5 PSU, 10 PSU and 5 PSU seawater. (B) Hemolymph osmolality response across treatments within the first 24 h. Error bars are expressed in standard error, and ‘**’ is *p* < 0.01.

There was a significant effect of salinity exposure (*n* = 160 total) on hemolymph osmolality for *H. sanguineus* (Fig. 4) over the course of seven days (*F* = 4.6371, *d.f.* = 7, *p* < 0.001), depending on salinity treatment (*F* = 12.0486, *d.f.* = 3, *p* ≪ 0.001), and interaction between time of exposure and treatment (*F* = 2.9242, *d.f.* = 21, *p* < 0.001). Salinity treatments were quite variable in hemolymph osmolality under 8 h of exposure, but did not significantly differ from one another for the 8-hour, 24-hour, and 48-hour treatments. At 72 h, hemolymph osmolality was significantly higher (one-way ANOVA, *F* = 7.055, *d.f.* = 3, *p* < 0.01, *n* = 20) in the 32 PSU treatment than both the 5 PSU (Tukey’s post-hoc test, *α* = 0.05, *p* < 0.01) and the 17.5 PSU (Tukey’s post-hoc test, *α* = 0.05, *p* < 0.05) treatments. At 168 h (7 days), hemolymph osmolality was significantly different across the four treatments (one-way ANOVA, *F* = 9.383, *d.f.* = 3, *p* < 0.001, *n* = 20) and the 5 PSU treatment was significantly lower in osmolality than all others (Tukey’s post-hoc test, *α* = 0.05, *p* < 0.01).

## Discussion

That survival declines for *H. sanguineus* over time for the 1 PSU treatment (Fig. 1) is noteworthy, but even prolonged periods of freshwater influx may not be effective in keeping *H. sanguineus* from surviving to establish a population, since the lowest survival rate after two weeks for these animals is still 65% at 1 PSU. Maintenance of internal hemolymph osmolality over 7 days (Fig. 4) by this species is consistent with its ability to survive. The point at which mortality began to increase in the 1 PSU treatment (∼ day 5), is consistent with the significantly lower internal hemolymph osmolality for the 5 PSU treatment of the hemolymph data only after 7 days of exposure. Work with other euryhaline crabs, like *Callinectes sapidus*, supports that this ability to maintain hemolymph osmolality within 12 h (Sommer & Mantel, 1988; Towle, 1997; Henry et al., 2002) helps the animal deal with estuarine osmotic stress. This study of *H. sanguineus* observed no change in hemolymph osmolality for 48 h, underscoring the survival ability of this crab and therefore its ability to invade new areas. This finding adds to earlier work which merely indicated that stress is induced at 15 PSU seawater for *H. sanguineus* (Depledge, 1984).

However, survival in a particular salinity is likely different from avoidance of suboptimal salinities*. H. sanguineus* individuals maintain a functional amount of Na^+^/K^+^ ATPase (Tsai & Lin, 2007; Hudson, 2011) to help them navigate this constantly changing environment, and likely uses behavioral strategies to avoid suboptimal salinities. There could be a major difference with one of these physiological characters and the physiological characters of the previously dominant intertidal crab, *Carcinus maenas*, which still has a depressed hemolymph osmolality at 7 days in low salinity (Siebers et al., 1982; Henry, Thomason & Towle, 2006), that may have impacted its competitive interaction with *H. sanguineus* and facilitated the latter’s invasion. As *H. sanguineus* maintains internal osmolality regardless of salinity treatment over short exposures, it may be more suited than competitors to the varying conditions of the intertidal zone. Therefore, it may be able to behaviorally maintain its shelter against competitors that leave under suboptimal salinity conditions, much like its congener *Hemigrapsus nudus* (Corotto & Holliday, 1996; McGaw, 2001). In the littoral zone, a change in salinity can occur during each tidal cycle and during a period of prolonged precipitation or spring melting, allowing these species to maintain territory if they are not behaviorally affected.

In the behavioral salinity choice data, a true choice of salinity was a far lesser signal (Fig. 2), and less informative, than the analysis of crabs leaving the starting level of salinity (Fig. 3). The decrease in likelihood of leaving as salinity increased is expected for optimal behavioral moderation of osmotic stress, but even the level of 51.3% of crabs leaving 5 PSU after 12 h is far less than the tidal cycle. This means that a large portion of crabs would remain in intertidal areas affected by regular salinity changes. This is an important finding with respect to metabolic stressors, as crustaceans have to switch to other physiological mechanisms, notably ammonia excretion (Shinji et al., 2009; Weihrauch, Morris & Towle, 2004), in order to maintain hemolymph osmolality at low salinities. The implication of males being more likely to leave the original salinity than females, likely due to differences in overall activity level between the sexes (Fig. 3), is that males are more likely to relocate into areas that are more suitable when salinity changes, whereas females will experience greater osmotic stress. Additionally, increased frequency of crabs leaving the starting salinity with increases in temperature means that *H. sanguineus* will be more likely to behaviorally respond to stressful salinities at higher temperatures than at those present during winter months, perhaps resulting in some seasonal differences in osmotic stress and mortality. Although the species can strongly osmoregulate at other salinities, those individuals relocating to full-strength salinity are likely to have an energetic advantage since there is less of a need for the excretion of ammonia (Weihrauch, Morris & Towle, 2004). This is also evident by the lower frequency of crabs leaving the starting salinity if they are starting in 35 PSU. This energetics argument is clear from recent work done in the congener *H. crenulatus*, which showed decreasing oxygen consumption and decreasing ammonia excretion as salinity increased (Urzúa & Urbina, 2017).

A large proportion of *H. sanguineus* individuals stayed in stressful starting salinities of 5 PSU (48.7% of the time did not leave) and 15 PSU (69.1% of the time did not leave), indicating that *H. sanguineus* should maintain territory by withstanding fluctuations in salinity that happen with rain events compared to other species. Such an inter-species comparison merits further study (Lucu & Towle, 2003; Tsai & Lin, 2007). The low likelihood of moving under stressful salinities may mean that this is a common trait to the genus, like their congener *H. nudus* (McGaw, 2001), and could result in faster geographic expansion and increased invasiveness of multiple members of the *Hemigrapsus* genus by decreasing exposure to predators (Jones & Shulman, 2008). These behavioral differences may be part of what is responsible for the more subtidal distribution of *C. maenas* observed in previous work (Hudson, Reagan & Crivello, 2016), not seen in the intertidal zone in estuarine areas where it does not overlap with *H. sanguineus* (Behrens Yamada & Gillespie, 2008; Amaral et al., 2009). Decreased desiccation of the smaller *H. sanguineus* using microhabitats in intertidal cobble fields when compared to the larger *C. maenas* (Altieri et al., 2010) may also have contributed to this intertidal dominance. This is also true for the mud crab *Eurypanopeus depressus* in intertidal oyster reefs (Grant & McDonald, 1979).

Physiological responses will continue to be useful in models to predict future invasions and the likely finer-scale distributions and competitive interactions in a new environment (Kneib, 1984; Zacherl, Gaines & Lonhart, 2003; Kimball et al., 2004; Rudnick et al., 2005; Herborg et al., 2007). Biological invasions continue worldwide with increasing human commerce (Pimentel, Zuniga & Morrison, 2005) so predicting a species’ probable impact by utilizing behavioral along with physiological characters synthesized with ecological and biogeographical theory will help facilitate our understanding of these processes. Behavior is becoming more prevalent as an explanation for invasion success (Weis, 2010), and this study adds to our understanding of how this invader’s distribution and use pattern arises from its physiology and behavior. Combining large-scale physical models as done for *H. sanguineus* in the Gulf of Maine (Delaney, Edwards & Leung, 2012) with small-scale estuarine behavior will offer a far higher resolution to spatial prediction. As invasions often gain a foothold on a small scale, the overall picture must include how the species in question interacts with these parameters on the local scale, in order to more accurately predict invasion success.

## Conclusions

As survival is high in this crab under low salinity conditions, freshwater input into an estuary will probably not greatly affect survival of populations of this species. The findings here indicate an advantage of *H. sanguineus* in surviving stressful changes in salinity during those periods, so more founding members should survive and therefore be more likely to establish in areas where it is introduced. The level at which *H. sanguineus* maintains its internal hemolymph osmolality, along with its high survival rate in a broad salinity range in this study, highlight its osmoregulatory character. However, as the energetic demands of this animal become more variable as temperature increases (Jungblut, 2017), it is important to investigate the interactive effect of seasonal salinity change on likely distribution.

The genus *Hemigrapsus* includes two prominent invaders in Europe and North America, as *H. takanoi* demonstrates a wide salinity tolerance (Shinji et al., 2009) and invaded Europe (originally misidentified as *H. penicillatus*) (Gollasch, 1998; Asakura & Watanabe, 2005) shortly followed by *H. sanguineus* (d’Udekem d’Acoz & Faasse, 2002) the effects of the behavioral dominance of *H. sanguineus* for shelter (Hudson, Reagan & Crivello, 2016) with its ability to withstand salinity changes give it a unique ability to maintain valuable intertidal shelter from predators and competitors during changes in tides and freshwater events. This in combination with broad salinity tolerance and preferences provide the opportunity for it to outlast competitors for shelter and food when exposed to suboptimal salinities.

##  Supplemental Information

10.7717/peerj.5446/supp-1 Figure S1 Survivorship over time at each salinity treatment The survivorship over time, or the survival function (dotted line), of *H. sanguineus* by salinity categories (1 PSU = red; 5 PSU = yellow; 10 PSU = green; 15 PSU = cyan; 35 PSU = blue) (top). Shaded areas represent the 95% confidence bands estimated for the salinity-specific non-parametric Kaplan–Meier survival functions. Plus signs (+) on each survival function indicate when an individual was last observed alive (i.e., right-censored). The frequency table tracks the cumulative number of right-censored individuals for each survival function over time (bottom). For greater interpretation, salinity-specific survival functions and associated 95% confidence bands are displayed individually (right).

10.7717/peerj.5446/supp-2 Figure S2 Total percent choice results of *H. sanguineus* for salinities at 20 °C These are listed by treatment: 5 PSU ×15 PSU (A: Male, B: Female, C: Total), 15 PSU ×35 PSU (D: Male, E: Female, F: Total), 5 PSU ×35 PSU (G: Male, H: Female, I: Total). Male (Left Column), Female (Middle Column), and Compiled (Right Column) data are shown, with pooled acclimation. Behavioral preference experiments indicated a significant preference (*χ*^2^ = 5.88, *d*.*f*. = 1, *p* < 0.05, *n* = 75) of *H. sanguineus* for 35 PSU over 5 PSU seawater at 20 °C regardless of acclimation (I), which persisted when data were analyzed by sex, as males (G) also showed this signal (*χ*^2^ = 9.52, *d*.*f*. = 1, *p* < 0.01, *n* = 42).

10.7717/peerj.5446/supp-3 Table S1 Impact of sex and salinity on survival ****Regression output coefficient table of the Cox proportional hazards regression model used to analyze the impact of salinity treatment and sex on overall survival of *H. sanguineus* (*n* = 160). Coefficient values for all categorical levels were estimated with respect to a reference level for each covariate, thus explaining the absence of the 1 PSU salinity treatment and male groups. Survival data for specimens in the 35 psu salinity group were removed from the analysis given no mortality events occurred and vastly skewed model results.

10.7717/peerj.5446/supp-4 Table S2 Survival comparisons by individual salinities Pairwise comparisons between five salinity treatment groups (1, 5, 10, 15, and 35 PSU) using the Peto & Peto modification of the Gehan-Wilcoxon test. Significance values were adjusted for multiple testing using the Benjamini-Hochberg procedure and bolded.

10.7717/peerj.5446/supp-5 Table S3 Survival comparisons by salinity treatment Pairwise comparisons between three broad salinity treatment categories (1 PSU; 5–15 [5, 10, 15] PSU; 35 PSU) using the Peto & Peto modification of the Gehan-Wilcoxon test. Significance values were adjusted for multiple testing using the Benjamini-Hochberg procedure and bolded.

10.7717/peerj.5446/supp-6 Table S4 Two-way ANOVA table for the effects of acclimation salinity and starting salinity Two-way ANOVA for comparison between the effects of acclimation salinity and starting salinity on frequency of those crabs leaving the starting salinity. Significant values (*α* < 0.05) are bolded, trends are in italics.

10.7717/peerj.5446/supp-7 Table S5 Two-way ANOVA table for the effects of acclimation salinity and sex Two-way ANOVA for comparison between the effects of acclimation salinity and sex on frequency of those crabs leaving the starting salinity. Significant values (*α* < 0.05) are bolded, trends are in italics.

10.7717/peerj.5446/supp-8 Table S6 Two-way ANOVA table for the effects f starting salinity and temperature Two-way ANOVA for comparison between the effects of starting salinity and temperature on the frequency of those crabs leaving the starting salinity. Significant values (*α* < 0.05) are bolded, trends are in italics.

10.7717/peerj.5446/supp-9 Table S7 Two-way ANOVA for the effects of sex and temperature Two-way ANOVA for comparison between the effects of sex and temperature on frequency of those crabs leaving the starting salinity. Significant values (*α* < 0.05) are bolded, trends are in italics.

10.7717/peerj.5446/supp-10 Supplemental Information 1 Survival of *Hemigrapsus sanguineus* at different treatment salinities Each data point represents the number of crabs left at a given salinity after each day, up to 14 days.

10.7717/peerj.5446/supp-11 Supplemental Information 2 Crab binary preference experiment data for different salinity pairs Each data point represents an individual crab’s behavior after 12 hours for the three experiment types (5 PSU ×35 PSU, 5 PSU ×15 PSU, or 15 PSU ×35 PSU), including whether it left the starting salinity, its initial acclimation salinity, the temperature of the experiment, and its sex.

10.7717/peerj.5446/supp-12 Supplemental Information 3 Hemolymph osmolality for *Hemigrapsus sanguineus* after exposure to different salinities over the course of 7 days Each data point represents the hemolymph osmolality measured for an individual *H. sanguineus* crab, with information about how long the animal was exposed and what salinity treatment the animal was subjected to.

## Additional Information and Declarations

### Field Study Permissions

The following information was supplied relating to field study approvals (i.e., approving body and any reference numbers): Field collections were approved by Connecticut Department of Environmental Protection (Scientific Collector’s Permits # SC-06040 and SC-09015).

### Data Availability

The following information was supplied regarding data availability: The raw data are provided in the Supplemental Files.

## References

[ref-1] Altieri AH, Van Wesenbeeck BK, Bertness MD, Silliman BR (2010). Facilitation cascade drives positive relationship between native biodiversity and invasion success. Ecology.

[ref-2] Amaral V, Cabral HN, Jenkins S, Hawkins S, Paula J (2009). Comparing quality of estuarine and nearshore intertidal habitats for *Carcinus maenas*. Estuarine, Coastal and Shelf Science.

[ref-3] Ameyaw-Akumfi C, Naylor E (1987). Spontaneous and induced components of salinity preference behaviour in *Carcinus maenas*. Marine Ecology Progress Series.

[ref-4] Asakura A, Watanabe S (2005). *Hemigrapsus takanoi*, new species, a sibling species of the common Japanese intertidal crab *H. penicillatus* (Decapoda: Brachyura: Grapsoidea). Journal of Crustacean Biology.

[ref-5] Barnes R (1967). The osmotic behaviour of a number of grapsoid crabs with respect to their differential penetration of an estuarine system. The Journal of Experimental Biology.

[ref-6] Behrens Yamada S, Gillespie G (2008). Will the European green crab (*Carcinus maenas*) persist in the Pacific Northwest?. ICES Journal of Marine Science.

[ref-7] Benoît HP, Capizzano CW, Knotek RJ, Rudders DB, Sulikowski JA, Dean MJ, Hoffman W, Zemeckis DR, Mandelman JW (2015). A generalized model for longitudinal short- and long-term mortality data for commercial fishery discards and recreational fishery catch-and-releases. ICES Journal of Marine Science: Journal du Conseil.

[ref-8] Brousseau DJ, Baglivo JA, Filipowicz A, Sego L, Alt C (2002). An experimental field study of site fidelity and mobility in the Asian shore crab, *Hemigrapsus sanguineus*. Northeastern Naturalist.

[ref-9] Bulger AJ, Hayden BP, Monaco ME, Nelson DM, McCormick-Ray MG (1993). Biologically-based estuarine salinity zones derived from a multivariate analysis. Estuaries.

[ref-10] Burnett LE, Towle DW (1990). Sodium ion uptake by perfused gills of the blue crab *Callinectes sapidus*: effects of ouabain and amiloride. Journal of Experimental Biology.

[ref-11] Case TJ, Taper ML (2000). Interspecific competition, environmental gradients, gene flow, and the coevolution of species’ borders. The American Naturalist.

[ref-12] Cieluch U (2004). Ontogeny of osmoregulatory structures and functions in the green crab *Carcinus maenas* (Crustacea, Decapoda). Journal of Experimental Biology.

[ref-13] Colnar AM, Landis WG (2007). Conceptual model development for invasive species and a regional risk assessment case study: the European Green Crab, *Carcinus maenas*, at Cherry Point, Washington, USA. Human and Ecological Risk Assessment: An International Journal.

[ref-14] Corotto FS, Holliday CW (1996). Branchial Na, K-ATPase and osmoregulation in the purple shore crab, *Hemigrapsus nudus* (Dana). Comparative Biochemistry and Physiology Part A: Physiology.

[ref-15] Cox DR (1972). Regression models and life-tables. Journal of the Royal Statistical Society. Series B (Methodological).

[ref-16] Cox DR, Oakes D (1984). Analysis of survival data.

[ref-17] Delaney DG, Edwards PK, Leung B (2012). Predicting regional spread of non-native species using oceanographic models: validation and identification of gaps. Marine Biology.

[ref-18] Depledge MH (1984). Cardiac activity in the intertidal crab *Hemigrapsus sanguineus* (De Haan). Asian Marine Biology.

[ref-19] d’Udekem d’Acoz C, Faasse M (2002). De huidige status van *Hemigrapsus sanguineus* (de Haan 1835) en *H. penicillatus* (de Haan 1835) in de noordelijke Atlantische Oceaan in het bijzonder in Nederland met opmerkingen over hun biologie (Crustacea Decapoda Brachyura). Het Zeepaard.

[ref-20] Engel DW (1977). Comparison of the osmoregulatory capabilities of two portunid crabs, *Callinectes sapidus* and *C. similis*. Marine Biology.

[ref-21] Epifanio CE (2013). Invasion biology of the Asian shore crab *Hemigrapsus sanguineus*: a review. Journal of Experimental Marine Biology and Ecology.

[ref-22] Felder DL (1978). Osmotic and ionic regulation in several Western Atlantic Callianassidae (Crustacea, Decapoda, Thalassinidea). The Biological Bulletin.

[ref-23] Fowler A, Forsström T, Von Numers M, Vesakoski O (2013). The North American mud crab *Rhithropanopeus harrisii* (Gould, 1841) in newly colonized Northern Baltic Sea: distribution and ecology. Aquatic Invasions.

[ref-24] Fowler AE, Gerner NV, Sewell MA (2011). Temperature and salinity tolerances of Stage 1 zoeae predict possible range expansion of an introduced portunid crab, *Charybdis japonica*, in New Zealand. Biological Invasions.

[ref-25] Gollasch S (1998). The Asian decapod *Hemigrapsus penicillatus* (de Haan, 1835) (Grapsidae, Decapoda) introduced in European waters: status quo and future perspective. Helgoländer Meeresuntersuchungen.

[ref-26] Gothland M, Dauvin JC, Denis L, Dufossé F, Jobert S, Ovaert J, Pezy JP, Rius ATous, Spilmont N (2014). Biological traits explain the distribution and colonisation ability of the invasive shore crab *Hemigrapsus takanoi*. Estuarine, Coastal and Shelf Science.

[ref-27] Gothland M, Dauvin J-C, Denis L, Jobert S, Ovaert J, Pezy J-P, Spilmont N (2013). Additional records and distribution (2011–2012)of *Hemigrapsus sanguineus* (De Haan, 1835) along the French coast of the English Channel. Management of Biological Invasions.

[ref-28] Grant J, McDonald J (1979). Desiccation tolerance of *Eurypanopeus depressus* (Smith) (Decapoda: Xanthidae) and the exploitation of microhabitat. Estuaries.

[ref-29] Gross WJ (1957). A behavioral mechanism for osmotic regulation in a semi-terrestrial crab. The Biological Bulletin.

[ref-30] Harrington DP, Fleming TR (1982). A class of rank test procedures for censored survival data. Biometrika.

[ref-31] Henry RP, Garrelts EE, McCarty MM, Towle DW (2002). Differential induction of branchial carbonic anhydrase and NA+/K+ ATPase activity in the euryhaline crab, *Carcinus maenas*, in response to low salinity exposure. Journal of Experimental Zoology.

[ref-32] Henry RP, Thomason KL, Towle DW (2006). Quantitative changes in branchial carbonic anhydrase activity and expression in the euryhaline green crab, *Carcinus maenas*, in response to low salinity exposure. Journal of Experimental Zoology Part A: Comparative Experimental Biology.

[ref-33] Herborg L-M, Rudnick DA, Siliang Y, Lodge DM, MacIsaac HJ (2007). Predicting the range of Chinese mitten crabs in Europe. Conservation Biology.

[ref-34] Hernández RM, Bückle RLF, Palacios E, Barón SB (2006). Preferential behavior of white shrimp *Litopenaeus vannamei* (Boone 1931) by progressive temperature–salinity simultaneous interaction. Journal of Thermal Biology.

[ref-35] Hobbs N-VS, Cobb JS, Thornber CS (2017). Conspecific tolerance and heterospecific competition as mechanisms for overcoming resistance to invasion by an intertidal crab. Biological Invasions.

[ref-36] Hudson DM (2011). Characteristics contributing to invasiveness of the Asian Shore Crab, *Hemigrapsus sanguineus*. D. Phil. thesis. Storrs.

[ref-37] Hudson DM, Reagan D, Crivello JF (2016). Community shelter use in response to two benthic decapod predators in the Long Island Sound. PeerJ.

[ref-38] Hulathduwa YD, Stickle WB, Brown KM (2007). The effect of salinity on survival, bioenergetics and predation risk in the mud crabs *Panopeus simpsoni and Eurypanopeus depressus*. Marine Biology.

[ref-39] Jansson B-O (1962). Salinity resistance and salinity preference of two oligochaetes *Aktedrilus monospermatecus* Knöllner and *Marionina preclitellochaeta* N.SP. from the interstitial fauna of marine sandy beaches. Oikos.

[ref-40] Jones PL, Shulman MJ (2008). Subtidal-intertidal trophic links: American lobsters [*Homarus americanus* (Milne-Edwards)] forage in the intertidal zone on nocturnal high tides. Journal of Experimental Marine Biology and Ecology.

[ref-41] Jungblut S (2017). Ecology and ecophysiology of invasive and native decapod crabs in the southern North Sea. D Phil. thesis.

[ref-42] Kaplan EL, Meier P (1958). Nonparametric estimation from incomplete observations. Journal of the American Statistical Association.

[ref-43] Kassambara A, Kosinski M (2017). https://cran.r-project.org/web/packages/survminer/index.html.

[ref-44] Kimball ME, Miller JM, Whitfield PE, Hare JA (2004). Thermal tolerance and potential distribution of invasive lionfish (*Pterois volitans/miles* complex) on the east coast of the United States. Marine Ecology Progress Series.

[ref-45] Kneib RT (1984). Patterns of invertebrate distribution and abundance in the intertidal salt marsh: causes and questions. Estuaries.

[ref-46] Koch HJ, Kitching JA (1954). Cholinesterase and active transport of sodium chloride through the isolated gills of the crab Eriocheir sinensis (M.Edw). Recent developments in cell biology.

[ref-47] Kotta J, Ojaveer H (2012). Rapid establishment of the alien crab *Rhithropanopeus harrisii* (Gould) in the Gulf of Riga. Estonian Journal of Ecology.

[ref-48] Kraemer GP, Sellberg M, Gordon A, Main J (2007). Eight-year Record of *Hemigrapsus sanguineus* (Asian Shore Crab) Invasion in Western Long Island Sound Estuary. Northeastern Naturalist.

[ref-49] Lagerspetz K, Mattila M (1961). Salinity reactions of some fresh- and brackish-water crustaceans. The Biological Bulletin.

[ref-50] Landschoff J, Lackschewitz D, Kesy K, Reise K (2013). Globalization pressure and habitat change: pacific rocky shore crabs invade armored shorelines in the Atlantic Wadden Sea. Aquatic Invasions.

[ref-51] Lohrer AM, Whitlatch RB (2002). Interactions among aliens: apparent replacement of one exotic species by another. Ecology.

[ref-52] Lohrer AM, Whitlatch RB, Wada K, Fukui Y (2000). Home and away: comparisons of resource utilization by a marine species in native and invaded habitats. Biological Invasions.

[ref-53] Lucu Č, Towle DW (2003). Na^+^+K^+^-ATPase in gills of aquatic crustacea. Comparative Biochemistry and Physiology Part A: Molecular & Integrative Physiology.

[ref-54] McDermott J (1998). The western Pacific brachyuran (*Hemigrapsus sanguineus*: Grapsidae), in its new habitat along the Atlantic coast of the United States: geographic distribution and ecology. ICES Journal of Marine Science.

[ref-55] McGaw IJ (2001). Impacts of habitat complexity on physiology: purple shore crabs tolerate osmotic stress for shelter. Estuarine, Coastal and Shelf Science.

[ref-56] McGaw IJ, Naylor E (1992a). Salinity preference of the shore crab *Carcinus maenas* in relation to coloration during intermoult and to prior acclimation. Journal of Experimental Marine Biology and Ecology.

[ref-57] McGaw IJ, Naylor E (1992b). The effect of shelter on salinity preference behaviour of the shore crab *Carcinus maenas*. Marine Behaviour and Physiology.

[ref-58] Neufeld GJ, Holliday CW, Pritchard JB (1980). Salinity adaption of gill Na, K-ATPase in the blue crab, Callinectes sapidus. Journal of Experimental Zoology.

[ref-59] O’Connor NJ (2014). Invasion dynamics on a temperate rocky shore: from early invasion to establishment of a marine invader. Biological Invasions.

[ref-60] Pimentel D, Zuniga R, Morrison D (2005). Update on the environmental and economic costs associated with alien-invasive species in the United States. Ecological Economics.

[ref-61] R Core Team (2017). https://www.R-project.org/.

[ref-62] Rabalais NN, Cameron JN (1985). Physiological and morphological adaptations of adult Uca subcylindrica to semi-arid environments. The Biological Bulletin.

[ref-63] Reisser CE, Forward RB (1991). Effect of salinity on osmoregulation and survival of a rhizocephalan parasite, *Loxothylacus panopaei*, and its crab host., *Rhithropanopeus harrisii*. Estuaries.

[ref-64] Rejmánek M, Simberloff D, Rejmánek M (2011). Invasiveness. Encyclopedia of biological invasions.

[ref-65] Roche DG, Torchin ME, Leung B, Binning SA (2009). Localized invasion of the North American Harris mud crab, Rhithropanopeus harrisii, in the Panama Canal: implications for eradication and spread. Biological Invasions.

[ref-66] Rudnick D, Veldhuizen T, Tullis R, Culver C, Hieb K, Tsukimura B (2005). A life history model for the San Francisco Estuary population of the Chinese mitten crab, *Eriocheir sinensis* (Decapoda: Grapsoidea). Biological Invasions.

[ref-67] Shinji J, Strüssmann CA, Wilder MN, Watanabe S (2009). Short-term responses of the adults of the common Japanese intertidal crab, *Hemigrapsus takanoi* (Decapoda: Brachyura: Grapsoidea) at different salinities: osmoregulation, oxygen consumption, and ammonia excretion. Journal of Crustacean Biology.

[ref-68] Siebers D, Leweck K, Markus H, Winkler A (1982). Sodium regulation in the shore crab *Carcinus maenas* as related to ambient salinity. Marine Biology.

[ref-69] Singer JD, Willett JB (2003). Applied longitudinal data analysis: modeling change and event occurrence.

[ref-70] Sommer MJ, Mantel LH (1988). Effect of dopamine, cyclic AMP, and pericardial organs on sodium uptake and Na/K-ATPase activity in gills of the green crab *Carcinus maenas* (L). Journal of Experimental Zoology.

[ref-71] Teal JM (1958). Distribution of fiddler crabs in Georgia salt marshes. Ecology.

[ref-72] Therneau T (2015). http://cran.r-project.org/package=survival.

[ref-73] Thomas NJ, Lasiak TA, Naylor E (1981). Salinity preference behaviour in *Carcinus*. Marine Behaviour and Physiology.

[ref-74] Towle DW (1997). Molecular approaches to understanding salinity adaptation of estuarine animals. American Zoologist.

[ref-75] Towle DW, Kays WT (1986). Basolateral localization of Na^+^ + K^+^-ATPase in gill epithelium of two osmoregulating crabs. *Callinectes sapidus and *Carcinus maenas**. Journal of Experimental Zoology.

[ref-76] Tsai J-R, Lin H-C (2007). V-type H^+^-ATPase and Na^+^, K^+^-ATPase in the gills of 13 euryhaline crabs during salinity acclimation. Journal of Experimental Biology.

[ref-77] Urbina M, Paschke K, Gebauer P, Chaparro OR (2010). Physiological energetics of the estuarine crab *Hemigrapsus crenulatus* (Crustacea: Decapoda: Varunidae): responses to different salinity levels. Journal of the Marine Biological Association of the United Kingdom.

[ref-78] Urzúa Á, Urbina MA (2017). Ecophysiological adaptations to variable salinity environments in the crab *Hemigrapsus crenulatus* from the Southeastern Pacific coast: sodium regulation, respiration and excretion. Comparative Biochemistry and Physiology Part A: Molecular & Integrative Physiology.

[ref-79] Van den Brink AM, Wijnhoven S, McLay CL (2012). Competition and niche segregation following the arrival of *Hemigrapsus takanoi* in the formerly *Carcinus maenas* dominated Dutch delta. Journal of Sea Research.

[ref-80] Weihrauch D, Morris S, Towle DW (2004). Ammonia excretion in aquatic and terrestrial crabs. Journal of Experimental Biology.

[ref-81] Weis JS (2010). The role of behavior in the success of invasive crustaceans. Marine and Freshwater Behaviour and Physiology.

[ref-82] Williams AB, McDermott JJ (1990). An eastern United States record for the western Indo-Pacific crab, *Hemigrapsus sanguineus* (Crustacea: Decapoda: Grapsidae). Proceedings of the Biological Society of Washington.

[ref-83] Wolf B, Kiel E, Hagge A, Krieg H-J, Feld CK (2009). Using the salinity preferences of benthic macroinvertebrates to classify running waters in brackish marshes in Germany. Ecological Indicators.

[ref-84] Young AM (1978). Desiccation tolerances for three hermit crab species *Clibanarius vittatus* (Bosc), *Pagurus pollicaris* Say and *P. longicarpus* Say (Decapoda, Anomura) in the North Inlet Estuary, South Carolina, USA. Estuarine and Coastal Marine Science.

[ref-85] Young AM (1979). Osmoregulation in three hermit crab species, *Clibanarius vittatus* (Bosc), *Pagurus longicarpus* Say and *P. pollicaris* Say (Crustacea: Decapoda; Anomura). Comparative Biochemistry and Physiology Part A: Physiology.

[ref-86] Zacherl D, Gaines SD, Lonhart SI (2003). The limits to biogeographical distributions: insights from the northward range extension of the marine snail, *Kelletia kelletii* (Forbes, 1852). Journal of Biogeography.

