# Supplementary figures and images for: Physiological and behavioral response of the Asian shore crab, Hemigrapsus sanguineus, to salinity: implications for estuarine distribution and invasion
Source: PeerJ. 2018 Aug 14;6:e5446. doi: 10.7717/peerj.5446 (PMC6097503; doi:10.7717/peerj.5446)

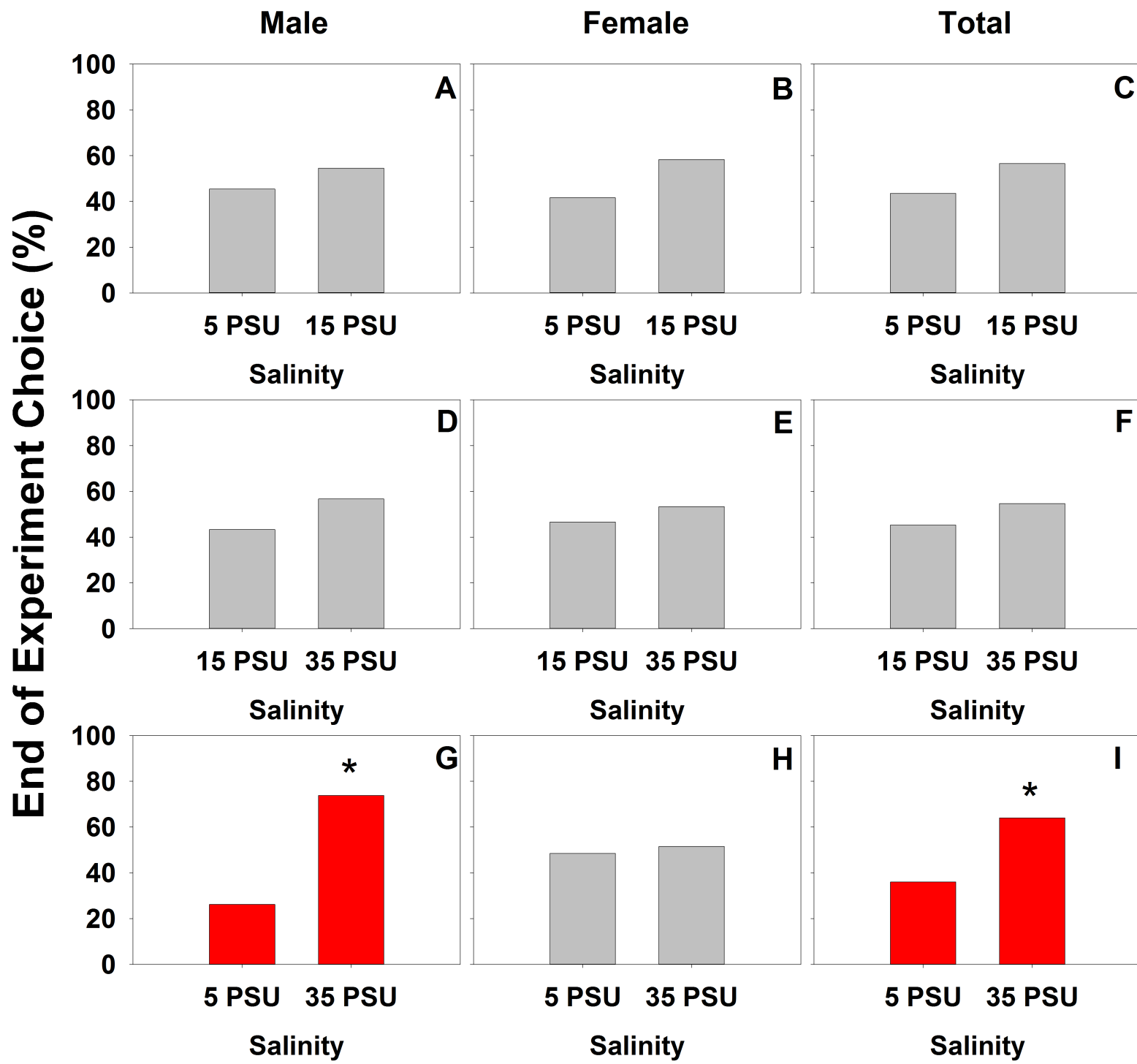

Supplement: Figure S2 — These are listed by treatment: 5 PSU ×15 PSU (A: Male, B: Female, C: Total), 15 PSU ×35 PSU (D: Male, E: Female, F: Total), 5 PSU ×35 PSU (G: Male, H: Female, I: Total). Male (Left Column), Female (Middle Column), and Compiled (Right Column) data are shown, with pooled acclimation. Behavioral preference experiments indicated a significant preference (χ2 = 5.88, d.f. = 1, p < 0.05, n = 75) of H. sanguineus for 35 PSU over 5 PSU seawater at 20 °C regardless of acclimation (I), which persisted when data were analyzed by sex, as males (G) also showed this signal (χ2 = 9.52, d.f. = 1, p < 0.01, n = 42). [file peerj-06-5446-s002.pdf]
