# Supplementary material for: Physiological and behavioral response of the Asian shore crab, Hemigrapsus sanguineus, to salinity: implications for estuarine distribution and invasion
Source: PeerJ. 2018 Aug 14;6:e5446. doi: 10.7717/peerj.5446 (PMC6097503; doi:10.7717/peerj.5446)
Supplement: Table S1 — Regression output coefficient table of the Cox proportional hazards regression model used to analyze the impact of salinity treatment and sex on overall survival of H. sanguineus (n = 160). Coefficient values for all categorical levels were estimated with respect to a reference level for each covariate, thus explaining the absence of the 1 PSU salinity treatment and male groups. Survival data for specimens in the 35 psu salinity group were removed from the analysis given no mortality events occurred and vastly skewed model results. [file peerj-06-5446-s003.docx]

|  | **Coef.** | **SE** | **HR** | **Lower CI** | **Upper CI** | ***z*-value** | ***p*-value** |
| --- | --- | --- | --- | --- | --- | --- | --- |
| Salinity |  |  |  |  |  |  |  |
| 5 PSU | -0.81247 | 0.46334 | 0.44376 | 0.17896 | 1.1004 | -1.754 | 0.080 |
| 10 PSU | -2.08011 | 0.75619 | 0.12492 | 0.02838 | 0.5499 | -2.751 | 0.006 |
| 15 PSU | -2.10546 | 0.75634 | 0.12179 | 0.02766 | 0.5363 | -2.784 | 0.005 |
| Sex |  |  |  |  |  |  |  |
| Female | -0.09763 | 0.40047 | 0.90698 | 0.41373 | 1.9883 | -0.244 | 0.807 |

Footnote: Parameter estimates for each covariate are listed and include the regression coefficient (Coef.), standard error of the regression coefficient (SE), the exponentiated coefficient called the hazard ratio (HR), 95% confidence intervals for the hazard ratio (CI), the Wald statistics value (*z*-value), and overall statistical significance (*p*-value).
