# Supplementary material for: Physiological and behavioral response of the Asian shore crab, Hemigrapsus sanguineus, to salinity: implications for estuarine distribution and invasion
Source: PeerJ. 2018 Aug 14;6:e5446. doi: 10.7717/peerj.5446 (PMC6097503; doi:10.7717/peerj.5446)
Supplement: Table S2 — Pairwise comparisons between five salinity treatment groups (1, 5, 10, 15, and 35 PSU) using the Peto & Peto modification of the Gehan-Wilcoxon test. Significance values were adjusted for multiple testing using the Benjamini-Hochberg procedure and bolded. [file peerj-06-5446-s004.docx]

|  | 1 | 5 | 10 | 15 |
| --- | --- | --- | --- | --- |
| 5 | 0.124 | - | - | - |
| 10 | **0.004** | 0.130 | - | - |
| 15 | **0.004** | 0.130 | 0.995 | - |
| 35 | **0.004** | **0.014** | 0.172 | 0.172 |
