# Supplementary material for: Physiological and behavioral response of the Asian shore crab, Hemigrapsus sanguineus, to salinity: implications for estuarine distribution and invasion
Source: PeerJ. 2018 Aug 14;6:e5446. doi: 10.7717/peerj.5446 (PMC6097503; doi:10.7717/peerj.5446)
Supplement: Table S3 — Pairwise comparisons between three broad salinity treatment categories (1 PSU; 5–15 [5, 10, 15] PSU; 35 PSU) using the Peto & Peto modification of the Gehan-Wilcoxon test. Significance values were adjusted for multiple testing using the Benjamini-Hochberg procedure and bolded. [file peerj-06-5446-s005.docx]

|  | 1 | 5-10-15 |
| --- | --- | --- |
| 5-10-15 | **0.0001** | - |
| 35 | **0.0001** | **0.048** |
