# Supplementary material for: Physiological and behavioral response of the Asian shore crab, Hemigrapsus sanguineus, to salinity: implications for estuarine distribution and invasion
Source: PeerJ. 2018 Aug 14;6:e5446. doi: 10.7717/peerj.5446 (PMC6097503; doi:10.7717/peerj.5446)
Supplement: Table S4 — Two-way ANOVA for comparison between the effects of acclimation salinity and starting salinity on frequency of those crabs leaving the starting salinity. Significant values (α < 0.05) are bolded, trends are in italics. [file peerj-06-5446-s006.docx]

| **Source of Variance** | **Sum of Squares** | ***df*** | **F-Value** | ***p*-value** |
| --- | --- | --- | --- | --- |
| Acclimation Salinity | 1.501 | 1 | 7.5523 | **0.006** |
| Starting Salinity | 12.961 | 2 | 32.6151 | **3.33e-14** |
| Acclimation Salinity x Starting Salinity | 0.948 | 2 | 2.3855 | 0.093 |
| Residuals | 124.978 | 629 |  |  |
