# Supplementary material for: Physiological and behavioral response of the Asian shore crab, Hemigrapsus sanguineus, to salinity: implications for estuarine distribution and invasion
Source: PeerJ. 2018 Aug 14;6:e5446. doi: 10.7717/peerj.5446 (PMC6097503; doi:10.7717/peerj.5446)
Supplement: Table S5 — Two-way ANOVA for comparison between the effects of acclimation salinity and sex on frequency of those crabs leaving the starting salinity. Significant values (α < 0.05) are bolded, trends are in italics. [file peerj-06-5446-s007.docx]

| **Source of Variance** | **Sum of Squares** | ***df*** | **F-Value** | ***p*-value** |
| --- | --- | --- | --- | --- |
| Acclimation Salinity | 1.408 | 1 | 6.5553 | **0.011** |
| Sex | 3.054 | 1 | 14.2121 | **0.0002** |
| Acclimation Salinity x Sex | 0.260 | 1 | 1.2089 | 0.272 |
| Residuals | 135.574 | 631 |  |  |
